# Prediction of treatment outcome in burning mouth syndrome patients using machine learning based on clinical data

**DOI:** 10.1038/s41598-021-94940-9

**Published:** 2021-07-28

**Authors:** Moon-Jong Kim, Pil-Jong Kim, Hong-Gee Kim, Hong-Seop Kho

**Affiliations:** 1 Department of Oral Medicine, Gwanak Seoul National University Dental Hospital, Seoul, South Korea; 2 Biomedical Knowledge Engineering Laboratory, School of Dentistry, Seoul National University, Seoul, South Korea; 3 Department of Oral Medicine and Oral Diagnosis, School of Dentistry and Dental Research Institute, Seoul National University, 101, Daehak-ro, Jongno-gu, Seoul, 03080 South Korea; 4 Institute On Aging, Seoul National University, Seoul, South Korea

**Keywords:** Mucositis, Prognostic markers, Oral medicine

## Abstract

The purpose of this study is to apply a machine learning approach to predict whether patients with burning mouth syndrome (BMS) respond to the initial approach and clonazepam therapy based on clinical data. Among the patients with the primary type of BMS who visited the clinic from 2006 to 2015, those treated with the initial approach of detailed explanation regarding home care instruction and use of oral topical lubricants, or who were prescribed clonazepam for a minimum of 1 month were included in this study. The clinical data and treatment outcomes were collected from medical records. Extreme Gradient-Boosted Decision Trees was used for machine learning algorithms to construct prediction models. Accuracy of the prediction models was evaluated and feature importance calculated. The accuracy of the prediction models for the initial approach and clonazepam therapy was 67.6% and 67.4%, respectively. Aggravating factors and psychological distress were important features in the prediction model for the initial approach, and intensity of symptoms before administration was the important feature in the prediction model for clonazepam therapy. In conclusion, the analysis of treatment outcomes in patients with BMS using a machine learning approach showed meaningful results of clinical applicability.

## Introduction

Burning mouth syndrome (BMS) is a chronic pain disorder in the orofacial region characterized by persistent oral burning sensation or dysesthesia without abnormal oral mucosal findings^1^. Various oral mucosal lesions (e.g. oral lichen planus, oral candidiasis, etc.) and systemic diseases (e.g. anemia, diabetes, etc.) were related to the development of oral burning sensation (secondary type of BMS). After the exclusion of these etiological factors, the diagnosis of real primary type of BMS can be made. Oral burning pain in the tongue mucosa is the main symptom of BMS. Besides burning pain, xerostomia and taste disturbance are common comorbid symptoms in patients with BMS. Based on numerous recent evidence, the primary type of BMS is considered as a neuropathic pain condition and caused by neuropathic changes occurring in complex patterns at various levels of the neuraxis^2^. The management of BMS remain challenging and various treatment modalities have been applied.

Detailed instructions regarding the possible etiopathophysiology of BMS, controlling oral parafunctional habits and the use of oral topical lubricants are important in the initial stage of BMS management. This procedure is simple, has no side effects, and can protect the oral mucosa from irritation or microtrauma that may lead to subclinical inflammation and worsening of neuropathy associated with the oral burning sensation. Reportedly, these procedures, referred to as the ‘initial approach’, were effective for oral discomfort associated with BMS in previous studies^3–5^.

Because the pathophysiology of BMS is associated with neuropathic mechanisms, neuropathic medications have been used to relieve BMS symptoms. The efficacy of various neuropathic medications including anxiolytics, anticonvulsants, anti-depressants, and capsaicin for the management of BMS has been reported. Among the neuropathic medications, clonazepam is the accepted mainstay for the treatment of BMS. Clonazepam has a high affinity for the benzodiazepine GABA_A_ receptor complex, which is widely distributed in the central nervous system as well as peripheral organs and associated with the occurrence of oral burning pain^6,7^. Clonazepam also enhances the descending pain modulation pathway^6,8^. In a recent meta-analysis, clonazepam therapy reportedly was an effective treatment modality for BMS regardless of the administration method (topical or systemic)^9^.

However, because the BMS neuropathic mechanisms are not simple and the underlying pathophysiology is different in each individual patient, the same treatments are not effective for all BMS patients^10^. Therefore, predicting treatment outcome in an individual patient in the early stage would be important in terms of reducing trial and error and enabling effective management of BMS symptoms. Neurophysiological examinations including functional brain imaging that could identify the underlying neuropathic mechanisms of an individual patient may provide information regarding the choice of medication and treatment outcomes. However, routinely performing these neurophysiological examinations in a clinical setting is not practical.

Comprehensive clinical examination procedures, including oral examinations, interviews with questionnaires, and diagnostic tests such as measurement of salivary flow rate and psychological screening, are routinely performed in the evaluation of patients with BMS. Clinical information from these procedures is readily available, and it has been proposed that the underlying neuropathic mechanisms could be partly estimated based on these clinical data^11,12^. In our two previous studies, the outcome predictors of BMS treatments were investigated based on clinical characteristics using classical statistical methods^4,13^. However, because BMS is a multifactorial condition and affected by the complex interactions of various clinical variables, predicting treatment outcome only with predictors reported in previous studies is difficult.

Recently, the machine learning approach has attracted attention as a new tool for recognizing and analyzing complex interaction patterns. The machine learning approach has the advantage of evaluating all potential predictors simultaneously to detect complex high dimensional interactions that might affect treatment outcomes. Through these processes, the machine learning approach can not only confirm the effect of individual clinical variables but also predict treatment outcome at the individual patient level by identifying data patterns of clinical variables. The purpose of this study is to apply a machine learning approach to predict whether patients with BMS respond to the initial approach and clonazepam therapy based on clinical data.

## Results

### Changes in the intensity of oral symptoms

**Figure 1:**
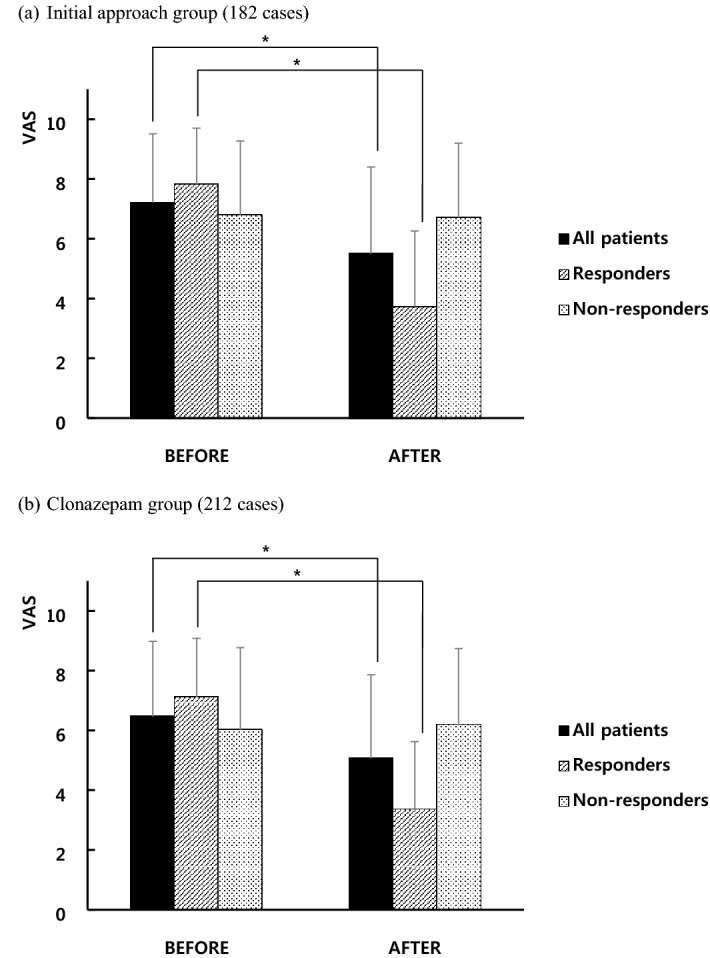
VAS scores of oral symptoms before and after (**a**) the initial approach and (**b**) clonazepam therapy. The error bars represent standard deviation (SD). VAS, visual analog scale. * *P* < 0.001.

The mean VAS scores of oral symptoms before and after treatments are shown in Fig. 1. The mean VAS scores of oral symptoms at baseline in the initial approach and clonazepam groups were 7.41 ± 2.15 and 7.53 ± 1.98, respectively. Significant differences were not observed in mean baseline VAS scores between responder and non-responder subgroups in both groups (data not shown). The mean VAS scores of all patients in both groups were significantly decreased after both the initial approach (5.50 ± 2.90) and clonazepam therapy (5.56 ± 2.80) compared to just before these treatments (initial approach group, 7.21 ± 2.30; clonazepam group, 6.46 ± 2.52) (*P* < 0.001), and these changes were only observed in the responder subgroup (*P* < 0.001).

### Prediction performance

Each machine learning model for the initial approach and clonazepam therapy was built using 131 features extracted from the clinical data of the initial approach and clonazepam groups. Table 1 shows the prediction performance of the two models. Both prediction models achieved an accuracy of 0.676 and 0.674, and ROC-AUC of 0.700 and 0.814, respectively. The model for the initial approach group prospectively identified 66.7% of responders and 68.2% of non-responders, and the model for the clonazepam group prospectively identified 41.2% of responders and 84.6% of non-responders.

**Table 1:** Prediction performance of the two models for the initial approach and clonazepam therapy.

	Initial approach (n = 182)	Clonazepam (n = 212)
Accuracy	0.676	0.674
AUC	0.700	0.814
Sensitivity	0.667	0.412
Specificity	0.682	0.846
PPV	0.588	0.636
NPV	0.750	0.688

AUC, area under receiver operating characteristic curve; PPV, positive predictive value; NPV, negative predictive value.

### Feature importance

**Figure 2:**
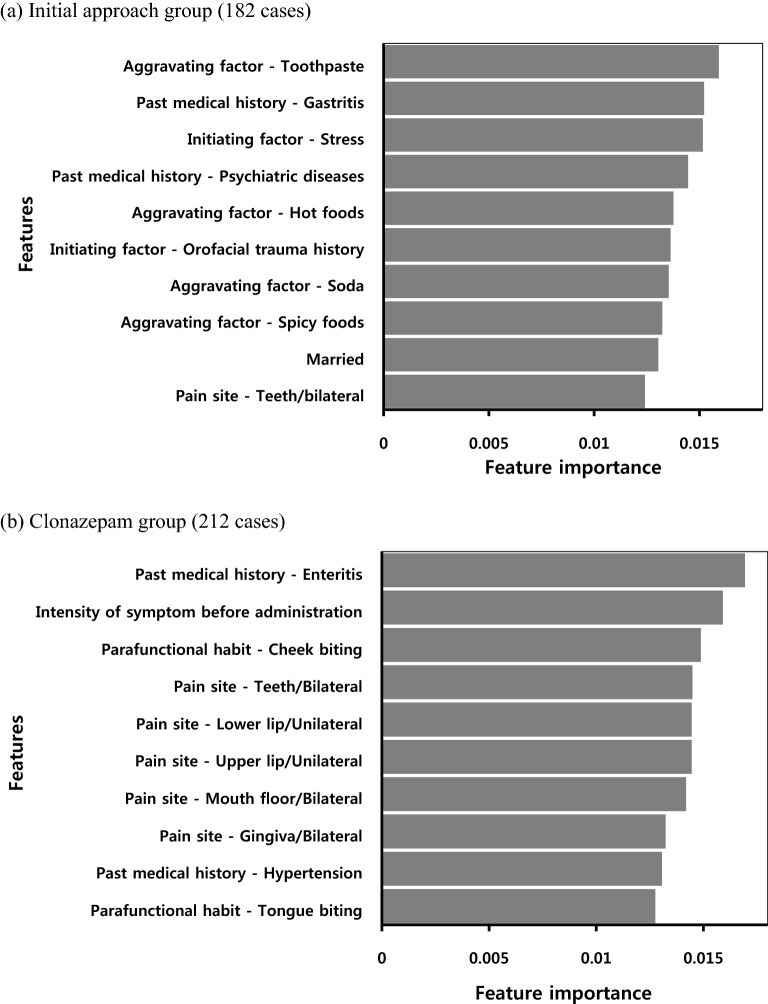
Top 10 feature importance for prediction models of (**a**) the initial approach and (**b**) clonazepam group datasets.

Figure 2 shows the top 10 feature importance results for the two prediction models trained on the initial approach and clonazepam groups. ‘Aggravating factor: toothpaste’ was the most important predictor of response in the prediction model for the initial approach group. In addition, among the 10 top feature importance results, the 4 most informative features were associated with aggravating factors (toothpaste, hot foods, soda, and spicy foods). Two psychologically related features (Initiating factor: stress and past medical history: psychiatric diseases) were also included in the top 10 features. ‘Past medical history: enteritis’ was the most important predictor, and ‘intensity of symptom before administration’ was the second most important predictor of response in the prediction model for the clonazepam group. Five features associated with pain site (teeth, lower and upper lips, mouth floor, and gingiva) and two features associated with parafunctional habits (cheek and tongue biting) were also included in the top 10 most informative features.

**Figure 3:**
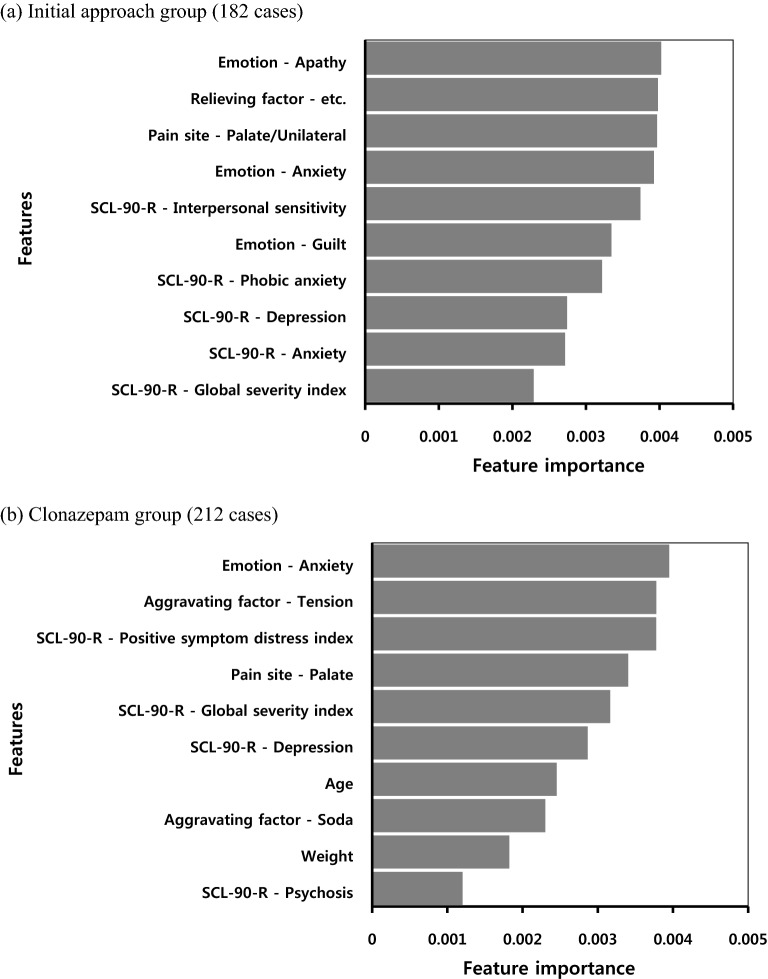
Lower 10 feature importance for prediction models of (**a**) the initial approach and (**b**) clonazepam group datasets. SCL-90-R, Symptom Checklist-90-Revised.

Figure 3 shows the lower feature importance results for the two prediction models trained on the initial approach and clonazepam groups, respectively. Five SCL-90-R items (global severity index, anxiety, depression, phobic anxiety, and interpersonal sensitivity) and three emotion-related features (guilt, anxiety, and apathy) were included in the lower 10 least informative features in the prediction model for the initial approach group. In the prediction model for the clonazepam group, four SCL-90-R items (psychosis, depression, global severity index, and positive symptom distress index) and one emotion-related feature (anxiety) were included in the lower 10 least informative features.

## Discussion

The primary therapeutic goal of BMS treatment is the relief of oral symptoms. Consequently, various neuropathic medications have been investigated for use in patients with BMS. Repetitive treatment failure can cause an increase negative expectations toward future treatments and worsening of psychological distress, consequently, adversely affecting the prognosis of treatments^14^. Therefore, selecting the appropriate neuropathic medication for patients with BMS is important. Unfortunately, the complexity and variety of underlying neuropathic changes in an individual patient with BMS renders the choice of medications difficult. Guidelines for BMS management have not been established to date and neuropathic medications are selected by trial and error.

To the best of our knowledge, this is the first study in which the relationship between clinical characteristics and treatment outcome in patients with BMS was analyzed using a machine learning approach. In the present study, the prediction models for the initial approach and clonazepam therapy achieved an accuracy of 0.676 and 0.674, respectively. The performance to predict whether patients will be responders or non-responders was limited in both models. The low sample number may be why the prediction performance in the models was limited. In addition, the clinical data used in this study may be insufficient to predict the treatment outcome. Adding the results of neurophysiological and/or additional diagnostic medicinal laboratory examinations to clinical data might be necessary for better prediction performance. Despite these limitations, the results of the present study may be meaningful because guidelines for medication therapies in BMS do not currently exist. In particular, the prediction model for the clonazepam group had a high specificity score (0.846) indicating the proposed model can be used as a screening tool to select non-responders to clonazepam therapy and to quickly move to the next step of treatment (change or addition of other neuropathic medications).

In the present study, the importance of features in both prediction models was calculated to identify clinical characteristics that influence treatment outcomes. Feature importance refers to the degree of usefulness of input features at predicting the outcome in a machine learning model. In the prediction model for the initial approach group, psychology-related features were among the top 10 most important predictors. This finding is consistent with previous studies^4,5^. Furthermore, several aggravating factors were the most important predictors. Psychological factors and aggravating factors are associated with irritation or microtrauma of the oral mucosa, and the initial approach may contribute to the relief of oral symptoms by avoiding the aggravating factors and supplementing the lubrication and protective function on the oral mucosa^3,4^. Conversely, intensity of symptom before treatment was one of the top 10 most important predictors in the prediction model for the clonazepam group, which was also consistent with the results from previous studies^5,13^. However, determining how each feature affects the treatment outcome by analyzing only the feature importance was not possible. Therefore, further studies in which the important predictors described in the present study are investigated regarding their effects on the treatment outcomes are necessary.

Notably, several SCL-90-R items were involved in the lower 10 least informative features in both prediction models, which is contradictory to previous studies using classical statistical methods^4,5,13^. The inconsistency between the results of the present study and previous studies may be associated with low prediction performance of the models. Furthermore, another feature may have been selected that has a similar but more meaningful information. For example, ‘past medical history: depression’ showed higher importance than SCL-90-R: depression in both models.

The present study had several limitations that should be addressed in future research. First, the number of subjects was not large enough to achieve sufficient model performance. Due to the prevalence of primary BMS, collecting data on a large scale is difficult. To overcome the sample size problem and improve the model performance to a level of clinical usefulness, multi-center research and/or the addition of other orofacial neuropathic pain conditions may be helpful. Second, all collected clinical features were used to construct prediction models in the present study. The values of clinical features that are not associated with treatment outcomes could adversely influence the predicted results of the models. Third, the treatment period to evaluate the response was only 1 month, which may be insufficient for the treatment to exert its full efficacy.

In conclusion, the performance of prediction models for the initial approach or clonazepam therapy constructed by machine learning was limited. However, due to the lack of data on treatment guidelines for BMS, prediction of treatment response based on a machine learning approach could be helpful. Further well-designed machine learning studies based on the large-scale dataset are necessary to develop a prediction model that reaches a clinically applicable level.

## Materials and methods

### Participants and data collection

This study is a retrospective study based on clinical data of participants who were selected from 420 patients with primary type of BMS visiting the Department of Oral Medicine, Seoul National University Dental Hospital from January 01, 2006 to December 31, 2015. These patients were the same as those included in our earlier study^5^. All participants were evaluated using oral examination, panoramic radiography, interview with a questionnaire on BMS, measurement of whole salivary flow rates, and simplified psychological evaluation (Symptom Checklist-90-Revised, SCL-90-R) at the first visit. The BMS questionnaire included items on sociodemographic characteristics, factors associated with the onset of oral symptoms, duration of suffering, distribution and area, and diurnal pattern of oral symptoms, aggravating and relieving factors, systemic diseases, and oral parafunctional habits. The evaluation procedure details were described in our previous study^11^. For alleviation of oral symptoms of BMS, the initial approach was firstly provided to all participants. Neuropathic medications were prescribed to patients who did not respond sufficiently to the initial approach, and clonazepam was administered as the first line neuropathic medication in almost patients. To be eligible, the patients had to be treated with the initial approach or prescribed clonazepam for a minimum of 1 month. Among the 420 patients, 182 and 212 patients were included in the initial approach and clonazepam groups, respectively. In the clonazepam group, clonazepam was administered at a dosage of approximately 0.25–1.5 mg/day. A majority of patients in both groups were middle-ages or elderly females (Table 2).

**Table 2:** Sample size, age and gender ratio of both treatment groups used to build machine learning models, mean ± S.D., n (%).

Therapy		Sample size (%)	Age* (Years)	Gender ^†^	
				Male (%)	Female (%)
Initial approach	Responder	74 (40.7)	62.4 ± 8.4	5 (6.8)	69 (93.2)
	Non-responder	108 (59.3)	62.2 ± 10.9	9 (8.3)	99 (91.7)
	Total	182 (100)	62.3 ± 10.0	14 (7.7)	168 (92.3)
Clonazepam	Responder	85 (40.1)	60.8 ± 12.2	5 (5.9)	80 (94.1)
	Non-responder	127 (59.9)	61.6 ± 9.3	11 (8.7)	116 (91.3)
	Total	212 (100)	61.3 ± 10.6	16 (7.5)	196 (92.5)

Responder, subjects whose visual analog scale (VAS) score on oral symptoms decreased more than 2 points after 1 month of the treatment compared with before treatment.

Non-responder, subjects whose VAS scores of oral symptoms decreased less than 2 points or experienced symptom worsening after 1 month of the treatment compared with before treatment.

* *P*-values of Student’s t-test between the responders and non-responders in the initial approach and clonazepam groups were 0.913 and 0.614, respectively.

^†^ *P*-values of chi-square test with correction in the initial approach and clonazepam groups were 0.913 and 0.627, respectively.

The intensity of oral symptoms was measured using the visual analog scale (VAS, 0–10, with 10 representing the worst extreme of symptoms) at baseline and at every visit to assess the treatment response evaluated using the differences of VAS score between before and after each treatment. Based on the treatment response, the participants were divided into dichotomous outcome subgroups (responder or non-responder). The responder group was defined as a decrease in VAS score of more than 2 points after 1 month of treatment compared with before treatment. The patients with a decrease in VAS score of less than 2 points or symptom worsening were considered as non-responders. The number of responders and non-responders were 74 and 108 in the initial approach group and 85 and 127 in the clonazepam group, respectively (Table 2).

This study was approved by the Institutional Review Board of Seoul National University Dental Hospital (#ERI 19047). Exemption from the need to obtain informed consent from the participants was also approved by the Institutional Review Board. All procedures were performed in accordance with relevant guidelines and regulations.

### Data pre-processing and feature selection

The clinical features used in construction of the prediction model were collected from clinical data in patient medical records. Demographic characteristics of patients (age, sex, height, weight, marital status, and educational level), whole salivary flow rates, SCL-90-R results, factors associated with the onset of oral symptoms, past medical history, menopause, emotional status, aggravating and relieving factors, oral parafunctional habits, and duration, area, and diurnal patterns of oral symptoms were used as the clinical features. Among those features, multi-categorical features were converted to dummy features by creating vectors of one and zero. A log transformation was applied to the skewed features to ensure that the histogram of features could approximate more closely a Gaussian distribution. The features which had only one value were removed because of their singularity. Missing values of features were imputed using the SimpleImputer function with median strategy in scikit-learn^15^. Processed data were converted to a standard normal distribution by subtracting means and dividing standard deviation of each feature.

### Construction of prediction model by machine learning approach

Pre-processed data were applied to the principal component analysis (PCA) for feature extraction using the sklearn.decomposition.PCA library^16^. Among extracted feature by PCA, features with p-value are less than or equal to 0.1 were obtained using the t-test feature selection.

The dataset was split into 80% training and 20% test sets with stratified sampling based on dependent features to prevent imbalance of dependent features between training and test datasets. To overcome the disadvantage of imbalanced data, over-sampling using the Synthetic Minority Oversampling Technique (SMOTE) was applied to the minority cases of dependent features using imbalanced-learn library^17^.

Extreme Gradient-Boosted Decision Trees (XGBoost) was used for machine learning algorithms to obtain the best accuracy of classification model^18^. The fivefold cross validation repeated 10 times using different splits was conducted when the model was trained. To tune hyper-parameters for the XGBoost model, random search was applied with 300 iterations in each repeated cross validation^19^. The hyper-parameters to tune were learning rate, max depth, subsample, early stopping rounds, n_estimators, gamma, colsample bytree, reg_alpha, reg_lambda, and min_child_weight. The range of each parameter was (1e-3, 1e-1), (3, 8), (0.5, 1.0), (1, 5), (1000, 4000), (0.0, 0.1), (0.9, 1), (1e-6, 1e0), (1e-1, 1e2), and (0, 5), respectively.

### Feature importance calculation

Feature importance was calculated using feature-case matrix, case-PCA matrix, and feature importance vector from the XGBoost model. The feature-case matrix and case-PCA matrix were multiplied to obtain the feature-PCA matrix. The selected features were chosen from PCA columns of the feature-PCA matrix. By multiplying the feature-selected PCA matrix with the feature-importance vector, which reflects the importance level of each selected PCA feature from the hyperparameter-tuned XGBoost model, the importance level of each feature could be calculated in the model.
